# Experiences with Surgical treatment of chronic lower limb ulcers at a Tertiary hospital in northwestern Tanzania: A prospective review of 300 cases

**DOI:** 10.1186/1471-5945-12-17

**Published:** 2012-09-28

**Authors:** Fidelis Mbunda, Mabula D Mchembe, Phillipo L Chalya, Peter Rambau, Stephen E Mshana, Benson R Kidenya, Japhet M Gilyoma

**Affiliations:** 1 Department of Surgery, Catholic University of Health and Allied Sciences- Bugando, Mwanza, Tanzania; 2 Department of Surgery, Muhimbili University of Health and Allied Sciences, Dar Es Salaam, Tanzania; 3 Department of Pathology, Catholic University of Health and Allied Sciences- Bugando, Mwanza, Tanzania; 4 Department of Microbiology, Catholic University of Health and Allied Sciences- Bugando, Mwanza, Tanzania; 5 Department of Biochemistry and Molecular Biology, Catholic University of Health and Allied Sciences- Bugando, Mwanza, Tanzania

**Keywords:** Chronic lower limb ulcers, Patterns, Treatment outcome, Predictors of outcome, Tanzania

## Abstract

**Background:**

Chronic lower limb ulcers constitute a major public health problem of great important all over the world and contribute significantly to high morbidity and long-term disabilities. There is paucity of information regarding chronic lower limb ulcers in our setting; therefore it was necessary to conduct this study to establish the patterns and outcome of chronic lower limb ulcers and to identify predictors of outcome in our local setting.

**Methods:**

This was a descriptive prospective study of patients with chronic lower limb ulcers conducted at Bugando Medical Centre between November 2010 and April 2012. Ethical approval to conduct the study was sought from relevant authorities. Statistical data analysis was done using SPSS version 17.0 and STATA version 11.0.

**Results:**

A total of 300 patients were studied. Their ages ranged from 3 months to 85 years (median 32 years). The male to female ratio was 2:1. The median duration of illness was 44 days. Traumatic ulcer was the most frequent type of ulcer accounting for 60.3% of patients. The median duration of illness was 44 days. The leg was commonly affected in 33.7% of cases and the right side (48.7%) was frequently involved. Out of 300 patients, 212 (70.7%) had positive aerobic bacterial growth within 48 hours of incubation. *Pseudomonas aeruginosa* (25.5%) was the most frequent gram negative bacteria isolated, whereas gram positive bacteria commonly isolated was *Staphylococcus aureus* (13.7%). Twenty (6.7%) patients were HIV positive with a median CD4+ count of 350 cells/μl. Mycological investigation was not performed. Bony involvement was radiologically reported in 83.0% of cases. Histopathological examination performed in 56 patients revealed malignancy in 20 (35.7%) patients, of which malignant melanoma (45.0%) was the most common histopathological type. The vast majority of patients, 270 (90.0%) were treated surgically, and surgical debridement was the most common surgical procedure performed in 24.1% of cases. Limb amputation rate was 8.7%. Postoperative complication rate was 58.3% of which surgical site infection (77.5%) was the most common post-operative complications. The median length of hospital stay was 23 days. Mortality rate was 4.3%. Out of the two hundred and eighty-seven (95.7%) survivors, 253 (91.6%) were treated successfully and discharged well (healed). After discharge, only 35.5% of cases were available for follow up at the end of study period.

**Conclusion:**

Chronic lower limb ulcers remain a major public health problem in this part of Tanzania. The majority of patients in our environment present late when the disease is already in advanced stages. Early recognition and aggressive treatment of the acute phase of chronic lower limb ulcers at the peripheral hospitals and close follow-up are urgently needed to improve outcomes of these patients in our environment.

## Background

Chronic ulceration of the lower limb constitutes a major public health problem of great important all over the world and contributes significantly to high morbidity and long-term disabilities [1]. It is a stressful disease to those affected as well as their family and the community in general, and its impact on hospital resources is great due to prolonged hospitalization, high cost of health care, loss of productivity and reduced quality of life [1-3]. Lower limb ulceration presenting late may end up being treated by limb amputation and is associated with increased risk of recurrence and malignant change [3].

Globally, the prevalence of chronic lower limb ulcers in the community has been reported in literature to range from 1.9 to 13.1% [1-3]. In developed countries, chronic ulceration of the lower limb affects approximately 2% of the population [4]. In the United Kingdom, the prevalence of chronic lower limb ulcers in the adult population is 1% and the prevalence in the more than 65 years age group is 3–5% [4,5]. In the United States of America, approximately 6,000,000 new lower limb ulcer cases are reported each year and in Sweden, 4–5% of the population over the age of 80 years presents with this pathology. The annual cost for treating chronic lower limb ulceration patients globally is estimated at some $25 million [6,7].

In Tanzania, chronic lower limb ulceration continues to be one of the leading causes of morbidity and long term disabilities. The disease tends to affect the young, reproductive age group. Observation at Bugando Medical Centre shows; chronic lower extremity ulceration is the single commonest indication for admission reported in the surgical wards and the majority of patients present late with advanced disease [8]. The etiological patterns of lower extremity ulceration in most developing countries have been reported to differ from that in developed countries. While, most of lower limb ulceration in the Western population is related to vascular diseases such as venous and arterial disease; trauma, malignancies, diabetes mellitus and infections are the most common causes in developing countries [2,9,10].

The effective treatment and outcome of lower limb ulceration is highly dependent upon establishing the etiology of the ulceration and the identification of other associated conditions that may have an adverse effect on healing [11].

The majority of chronic lower limb ulcers are preventable and have a multifactorial etiology, therefore understanding the etiological pattern of this condition in our local setting will provides information that is important for accurate diagnosis, prediction of outcome and may help in hospital resource allocations and establishment of prevention strategies as well as treatment protocols [12].

The aim of this study was to describe the patterns and treatment outcome of chronic lower limb ulcers in our local setting and to identify factors predicting the outcome. The study provides basis for establishment of treatment guidelines as well as prevention strategies.

## Methods

### Study design and setting

This was a descriptive prospective hospital-based study of patients with chronic lower limb ulcers carried out at Bugando Medical Centre (BMC) in Northwestern Tanzania between November 2010 and February 2012. BMC is located in Mwanza city along the shore of Lake Victoria in the northwestern part of Tanzania. It is a tertiary care and teaching hospital for the Catholic University of Health and Allied Sciences- Bugando (CUHAS-Bugando) and other paramedics and has a bed capacity of 1000. BMC is one of the four largest referral hospitals in the country and serves as a referral centre for tertiary specialist care for a catchment population of approximately 13 million people from Mwanza, Mara, Kagera, Shinyanga, Tabora and Kigoma regions.

### Study subjects and procedures

The study included all patients with chronic lower limb ulcers of all age groups and both genders seen in the surgical wards and surgical outpatient clinics of BMC during the study period. Patients who failed to consent for the study, treatment (e.g. limb amputation) and HIV testing were excluded from the study.

Recruitment of patients to participate in the study was done at the Accident and Emergency department, surgical outpatient clinic and in the surgical wards. Patients were screened for inclusion criteria and those who met the inclusion criteria were offered explanations about the study and requested to consent before being enrolled into the study. Convenience sampling of patients who met the inclusion criteria was performed until the sample size was reached. The diagnosis of chronic lower limb ulcers was made by clinical history and physical examination and chronic lower limb ulcers was defined as defect in the skin on the lower extremities that remains unhealed for at least four or more weeks. Pus or pus swabs were obtained from the ulcer and transported to the laboratory within an hour of collection. In the laboratory, the specimens were registered in the log books and processed as per standard operative procedures. Bacterial identification was done using an in house biochemical panel [13]. Antibacterial susceptibility testing to various antibiotics was performed using disc diffusion methods as previously described [14,15]. In addition, blood was taken from all patients for random blood sugar testing and CD4 enumeration in HIV positive patients. HIV test was done using national algorithm of rapid test. Mycological investigation was not performed due to logistic problems. Biopsies from chronic lower limb ulcers were taken under sterile technique and specimens were transported in a formalin solution to the histopathology laboratory for processing.

All recruited patients were managed accordingly. The authors ensured that the study patients were receiving the appropriate treatment and supportive care as prescribed by the surgeon. Patients were followed up until discharge or death. After discharge patients were followed up at our surgical outpatient clinic for up to six months.

Data were collected using a pre-tested coded questionnaire. Data administered in the questionnaire included; patients characteristics (e.g. age, sex, premorbid illness, history of smoking and use of immunosuppressive drugs), causes of chronic lower limb ulcers, clinical pattern, investigations, treatment modalities and postoperative complications. Length of hospital stay (LOS) and mortality were recorded at the end of study period.

### Statistical data analysis

Statistical data analysis was done using SPSS software version 17.0 (SPSS, Inc, Chicago, IL) and STATA version 11.0. Data was summarized in form of proportions and frequent tables for categorical variables. Continuous variables were summarized using means, median, mode and standard deviation. P-values were computed for categorical variables using Chi – square (χ^2^) test and Fisher’s exact test depending on the size of the data set. Independent student t-test was used for continuous variables. Multivariate logistic regression analysis was used to determine predictor variables that are associated with outcome. Post-operative complications were entered into univariate and multivariate analysis after been categorized into presence or absence of post-operative complications. LOS was arbitrarily categorized as ≤14 and > 14 days. A p-value of less than 0.05 was considered to constitute a statistically significant difference.

### Ethical consideration

Ethical approval to conduct the study was obtained from the CUHAS-Bugando/BMC joint institutional ethic review committee before the commencement of the study. Informed consent was sought from each patient before being enrolled into the study.

## Results

**Figure 1:**
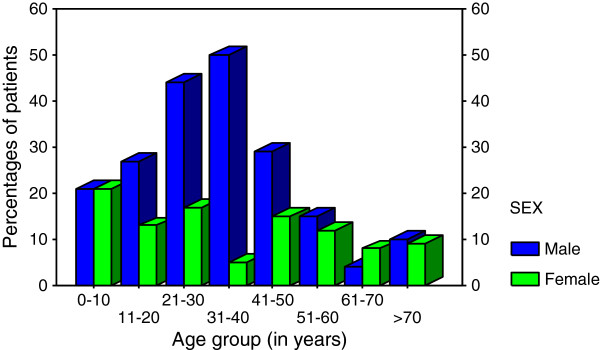
Sex distribution according to age group (In years).

During the period under study, a total of 312 patients with chronic lower limb ulcers were managed at Bugando Medical Centre. Of these, 12 patients were excluded from the study due failure to meet the inclusion criteria. Thus, 300 patients were studied. The ages of the study population ranged from 3 months to 85 years with a median of 32 years. The modal age group was 21–30 years. Out of 300 patients recruited into the study, two hundred (66.7%) were males and 100 (33.3%) were females. The male to female ratio was 2:1 with a male predominance in each age group (Figure 1).

Fifty-four (18.0%) patients presented with history of premorbid illness such as diabetes mellitus in 32 (59.3%), chronic pulmonary diseases in 8 (14.8%), hypertension in 6 (11.1%), peripheral vascular diseases in 4 (7.4%) and congenital cardiac diseases and obstructive jaundice in 2 (3.7%) patients each respectively. In this study, sixty-eight (22.7%) patients had history of cigarette smoking. There was no history of immunosuppressive drugs use or radiotherapy.

The median duration of illness was 44 days (range 31 to 3218 days). Traumatic ulcers were the most frequent type of ulcer accounting for 60.3% of patients. Road traffic accidents (RTAs) were the most common cause of traumatic ulcers accounting for 122 (67.4%) patients (Table 1). Seventy-three (59.8%) of RTAs were related to motorcycle injuries. Other causes of traumatic ulcers included burn in 45 (24.9%), falls in 8 (4.4%), gunshot injuries in 3(1.7%0, hit by falling object and sport injuries in 2(1.1%) and 1(0.6%) respectively. Table 1 shows distribution of study population according to the type of ulcers.

**Table 1:** **Distribution of study populationaccording to the typeof ulcers**

**Type of ulcers**	**Frequency**	**Percentage**
**Traumatic ulcers**	**181**	**60.3**
· Mechanical trauma	131	72.4
· Burns	50	27.6
**Infective ulcers**	**43**	**14.3**
· Osteomyelitis	30	69.8
· Tropical ulcer	6	13.9
· Cellulitis	5	11.6
· Others	2	4.7
**Metabolic ulcers**	**35**	**11.7**
· Diabetic ulcer	32	91.4
· Pellagra	3	8.6
**Neoplastic/malignant ulcers**	**20**	**6.7**
· Malignant melanoma	9	45.0
· Kaposi’s sarcoma	5	25.0
· Squamous cell carcinoma	4	20.0
· Others	2	10.0
**Vascular ulcers**	**11**	**3.7**
· Arterial ulcers	5	45.5
· Venous ulcers	4	36.4
· Mixed ulcers	2	18.2
**Neuropathic ulcers**	**8**	**2.7**
· Pressure sores	6	75.0
· Others	2	25.0
**Ulcerating skin lesions e.g.** Pyogenic Granulomatous	**2**	**0.7**

**Figure 2:**
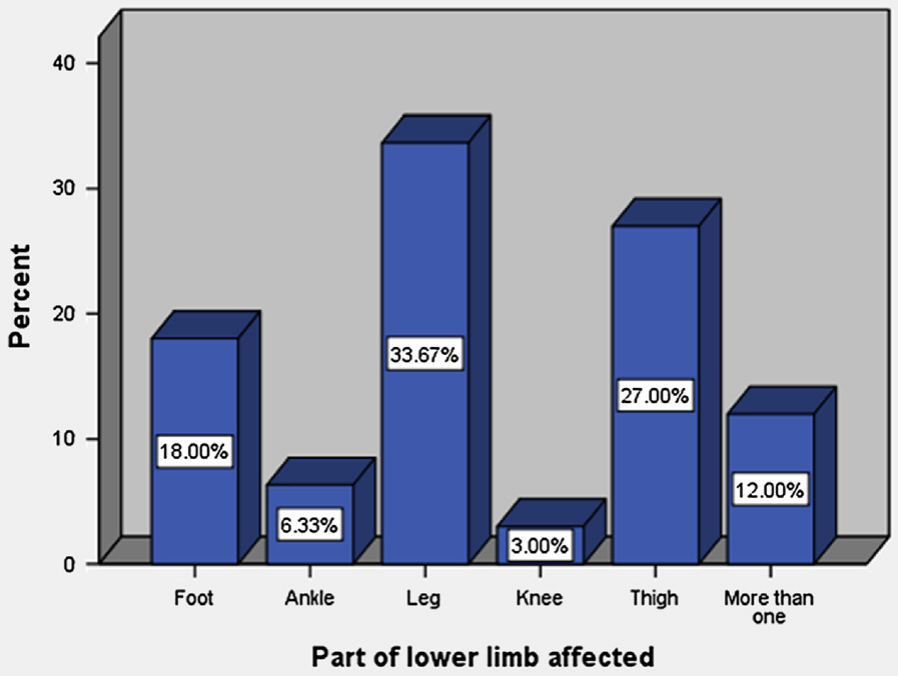
Part of lower limb affected.

The leg was commonly affected in 33.7% of cases (Figure 2) and the right, left and both limbs were involved in 146 (48.7%), 126 (42.0%) and 28 (9.3%) patients respectively. The size ulcers ranged from 2 to 30 cm with a median of 5 cm.

Out of 300 patients with CLLUs, 212 (70.7%) had positive aerobic bacterial growth within 48 hours of incubation. Of these, 10 (4.7%) patents had polymicrobial growth. *Pseudomonas spp*. (25.5%) and *Proteus spp*. (21.2%) were the most common bacteria isolated, while the least isolated bacteria were *Enterobacter spp* (2.8%) and *Enterococcus spp* (1.4%). Forty-one (19.3%) bacterial isolates were found to be Extended Spectrum Beta-Lactamases (ESBL) producers (i.e. resistant to first, second, third and fourth generation cephalosporins). Methicilin Resistant *Staphylococcus aureus* (MRSA) was detected in 23 out of 29 (79.3%) *Staphylococcus aureus* isolates.

In this study, twenty (6.7%) patients were HIV positive. Of these, 6 (30.0%) patients were known cases on ant-retroviral therapy (ARV) and the remaining 14 (70.0%) patients were newly diagnosed patients. Their CD 4+ count, available in 15 patients, ranged from 180 to 480 cells/μl (median = 350cells/μl). A total of two HIV patients (13.3%) had CD4+ count below 200 cells/μl and the remaining 13 patients (86.7%) had CD4+ count of ≥200 cells/μ.

Plain x-rays of the affected limbs were performed in 235 (78.3%) patients. Of these, 195 (83.0%) patients had abnormal x-ray findings including associated fractures, chronic osteomyelitis, bone tumors and others in 144 (73.9%), 47(24.1%), 3 (1.5%) and 1 (0.5%) patients respectively.

Doppler ultrasound of the affected limbs was done in 203 (67.7%) patients. Of these, only seventeen (8.4%) patients had abnormal Doppler ultrasound findings.

A total of 56 histopathological examinations were performed. Of these, 20 (35.7%) had a histopathologically proven malignancy, of which malignant melanoma was the most common histopathological type in 9 (45.0%) patients. This was followed by Kaposi’s sarcoma in 5 (25.0%), squamous cell carcinoma in 4 (20.0%), neurofibrosarcoma and liposarcoma in 1 (5.0%) patient each respectively.

**Figure 3:**
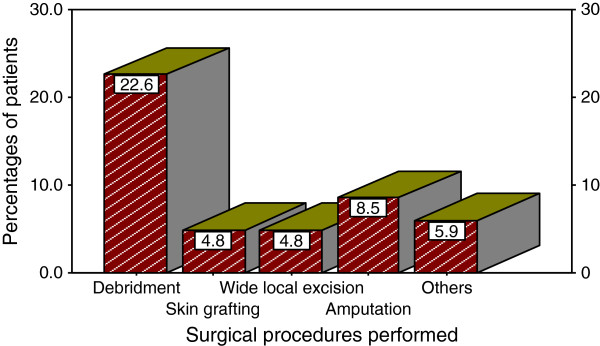
Distribution of patients according to surgical procedure performed.

A total of 287 (95.7%) patients were treated as inpatients and the remaining 13 (4.3%) patients were treated as outpatients. The vast majority of patients, 270 (90.0%) were treated surgically (Figure 3). The remaining 30 (10.0%) patients were treated conservatively (non-surgical approach) with daily dressing, antimicrobial agents, compression bandage, antibiotics.

**Figure 4:**
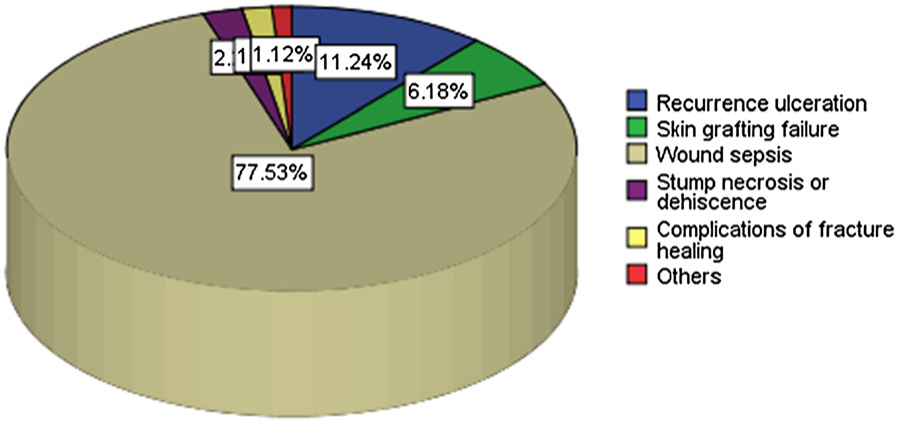
Distribution of patients according to postoperative complications.

A total of 178 post-operative complications were recorded in 175 (58.3%) patients. Of these, surgical site infection (77.5%) was the most common post-operative complications (Figure 4). Table 2 shows predictors of postoperative complications among patients with chronic lower limb ulcers according to univariate and multivariate logistic regression analysis

**Table 2:** **Predictors of Postoperative complicationsaccording to univariate andmultivariate logistic regression analysis**

**Predictor (Independent) variables**	**Post operative complications n (%)**		**Univariate analysis**			**Multivariate analysis**		
	**Absent**	**Present**	**OR**	**95% CI**	**P value**	**OR**	**CI**	**P value**
**Age**								
≤20	39(48.8)	41(51.3)						
>20	83(37.7)	137(62.3)	1.6	0.9-2.6	0.087	3.0	1.4-6.3	**0.004**
**Sex**								
Male	102(51.0)	98(49.0)						
Female	20(20.0)	80(80.0)	4.2	2.4-7.3	<0.001	4.3	2.1-8.7	**<0.001**
**HIV status**								
Positive	4(20.0)	16(80.0)						
Negative	118(42.1)	162(57.9)	2.9	0.9-8.9	0.062			
**CD4 count**								
<200 cells/μl	1(50.0)	1(50.0)						
≥200 cells/μl	3(23.1)	10(76.9)	1.3	0.2-2.7	0.082			
**Pre-morbid illness**								
Absent	11(20.4)	43(79.6)						
Present	111(45.1)	135(54.9)	3.2	1.6-6.5	0.001	0.4	0.2-1.0	**0.041**
**Tobacco smoking**								
Yes	30(44.1)	38(55.9)						
No	92(39.7)	140(60.3)	1.9	0.4- 5.4	0.897			
**Duration of illness**								
1/12-3/12	115(48.7)	121(51.3)						
3/12-1 year	3(7.1)	39(92.9)	12.4	3.7-41.1	<0.001	8.0	2.1-30.0	**0.002**
>1 year	4(18.2)	18(81.8)	4.3	1.4-13.0	0.010			
**Type of ulcer**								
Traumatic ulcer	107(59.1)	74(40.9)						
Vascular ulcer	4(36.4)	7(63.6)	2.5	0.7-9.0	0.150			
Neoplastic ulcer	4(20.0)	16(80.0)	5.8	1.9-18.0	0.002			
Infective ulcer	3(7.0)	40(93.0)	19.3	5.7-64.7	<0.001	17.6	4.7-66.0	**<0.001**
Metabolic ulcer	2(5.7)	33(94.3)	23.9	5.6-102.5	<0.001	8.4	1.8-39.4	**0.007**
Neuropathic ulcer	2(25.0)	6(75.0)	4.3	0.9-22.1	0.077			
Ulcerating skin disease	0(0.0)	2(100)	--	--	--			
**Ulcer size in cm**								
≤5	90(47.9)	98(52.1)						
>5-10	28(31.8)	60(68.2)	2.0	1.2-3.4	0.013			
>10	4(16.7)	20(83.3)	4.6	1.5-13.9	0.007			
**Mode of treatment**								
Conservative	14(36.8)	24(63.2)						
Surgical	108(41.2)	154(58.8)	0.8	0.4-1.7	0.608			

The overall length of hospital stay (LOS) ranged from 1 day to 180 days with a median of 23 days. The LOS for non-survivors ranged from 1 day and 42 days (median = 10 days). Table 3 shows predictors of LOS among patients with chronic lower limb ulcers according to univariate and multivariate logistic regression analysis

**Table 3:** **Predictors of length ofhospital stay (LOS) amongpatients with chronic lowerlimb ulcers according tounivariate and multivariate logisticregression analysis**

**Predictor (Independent) variables**	**LOS n (%)**		**Univariate analysis**			**Multivariate analysis**		
	**≤14**	**>14**	**OR**	**95% CI**	**P- value**	**OR**	**CI**	**P-value**
**Age**								
<20	14(17.5)	66(82.5)	1					
>20	45(20.4)	175(79.6)	1.1	0.4-1.6	0.570			
**Sex**								
Male	42(21.0)	158(79.0)	1					
Female	17(17.0)	83(83.0)	1.3	0.7-2.4	0.412			
**HIV status**								
Positive	2(10.0)	18(90.0)	1					
Negative	57(20.4)	223(79.6)	2.3	0.5-10.2	0.273			
**Pre-morbid illness**								
Absent	51(20.7)	195(79.3)	1					
Present	8(14.8)	46(85.2)	1.5	0.7-3.4	0.324			
**Duration of illness**								
1/12-3/12	47(19.9)	189(80.1)	1					
3/12-1 year	7(16.7)	35(83.3)	1.2	0.5-3.0	0.624			
>1 year	5(22.7)	17(77.3)	0.8	0.3-2.4	0.753			
**Type of ulcer**								
Traumatic ulcer	42(23.2)	139(76.8)	1					
Vascular ulcer	2(18.2)	9(81.8)	1.4	0.3-6.5	0.701			
Neoplastic ulcer	4(20.0)	16(80.0)	1.2	0.4-3.8	0.746			
Infective ulcer	6(14.0)	37(86.1)	1.9	0.7-4.7	0.189			
Metabolic ulcer	3(8.6)	32(91.4)	3.2	0.9-11.1	0.063	3.5	1.0-12.5	**0.056**
Neuropathic ulcer	1(12.5)	7(87.5)	2.1	0.3-17.7	0.489			
Ulcerating skin disease	1(50.0)	1(50.0)	0.3	0.01-4.9	0.401			
**Ulcer size in cm**								
≤5	38(20.2)	150(79.8)	1					
>5-10	17(19.3)	71(80.7)	1.1	0.6-2.0	0.862			
>10	4(16.7)	20(83.3)	1.3	0.4-3.9	0.682			
**Mode of treatment**								
Conservative	13(34.2)	25(65.8)	1					
Surgical	46(17.6)	216(82.4)	2.4	1.2-5.1	0.018	3.2	1.4-7.1	**0.005**
**Postoperative complications**								
Absent	26(21.3)	96(78.7)	1					
Present	33(18.5)	145(81.5)	1.2	0.7-2.1	0.553			

In this study, thirteen patients died giving a mortality rate of 4.3%. The causes of death were complications of diabetes mellitus (5 patients), HIV infection (4 patients) and advanced malignancy (2 patients). The cause of death was not established in 2 patients.

Out of the two hundred and eighty-seven (95.7%) survivors, 253 (91.6%) were treated successfully and discharged well (healed). Thirteen (4.5%) patients were discharged with permanent disabilities resulting from lower limb amputation and the remaining six (2.1%) patients were discharged home advised to continue with daily dressing at their nearby health facilities. Thirteen (4.5%) patients were treated as outpatients and two (0.7%) patients discharged themselves against medical advice.

## Discussion

In this review, chronic lower limb ulcers were in the third decade of life and tended to affect more males than females, with a male to female ratio of 2:1 which is comparable with other studies in developing countries [16,17]. Our demographic profile is in sharp contrast to what is reported in developed countries where the majority of the patients are in the sixth decade and above [17-19]. Male predominance in this age group may be due to their increased susceptibility to trauma which was found to be the major etiological agent of chronic lower limb ulcers in this study.

The presence of pre-morbid illness, such as diabetes mellitus, chronic obstructive pulmonary disease, arteriosclerosis, peripheral vascular disease, heart disease, and any conditions leading to hypotension, hypovolaemia, edema, and anemia has been reported elsewhere to have an effect on the outcome of patients with chronic lower limb ulcers [20,21]. Pre-morbid illnesses influence the healing process as a result of their influence on a number of bodily functions [20-23]. In the presence study, diabetes mellitus was the most common premorbid illness accounting for 59.3% of cases which is agreement with other studies in developing countries [21,22,24]. Diabetes mellitus is associated with delayed cellular response to injury, compromised cellular function at the site of injury, defects in collagen synthesis, and reduced wound tensile strength after healing. Diabetes-related peripheral neuropathy, reducing the ability to feel pressure or pain, contributes to a tendency to ignore pressure points and avoid pressure relief strategies [25].

In the present study, cigarette smoking was reported in 22.7% of cases which is in keeping with other studies [26,27]. Cigarette smoking has been reported to have an impact on wound healing through impairment of tissue oxygenation and local hypoxia via vasoconstriction [28]. Tobacco smoke has high concentration of carbon monoxide, which binds hemoglobin, forming carboxyhemoglobin. Carboxyhemoglobin binds to oxygen with high affinity and thereby interferes with normal oxygen delivery to hypoxic tissues [29].

The etiological pattern of chronic lower limb ulcers have been reported in literature to vary from one part of the world to another depending on the prevailing socio-demographic and environmental factors [2,9]. In Western societies, most chronic lower limb ulcers are due to vascular diseases, whereas in developing countries, trauma, infections, malignancies and poorly controlled diabetes remain the most common causes of chronic lower limb ulceration [2,9,10]. In the present study, traumatic ulcers secondary to road traffic accidents were the most common type of chronic lower limb ulcers accounting for more than sixty percent of cases, which is in keeping with other studies done in developing countries [9,10,20,21]. High incidence of traumatic ulcers secondary to road traffic accidents may be attributed to recklessness and negligence of the driver, poor maintenance of vehicles, driving under the influence of alcohol or drugs and complete disregard of traffic laws.

In agreement with other studies in developing countries [3,24], the majority of patients in the present study presented late to hospital with advanced and complicated chronic lower limb ulcers which may end up being treated by limb amputation with increased risk of recurrence and malignant change. Late presentation in this study may be attributed to poor economic capabilities in cost shared healthcare systems, inadequate knowledge of self-care and socio-cultural reasons. Other contributing factors for late presentation include attempts at home surgery, trust in faith healers, poor management of acute lower limb ulcers and delayed referral in most health centers and peripheral hospitals.

As reported in other studies [30,31], the leg was the most frequent anatomical site affected in our series and the right side was frequently involved. We could not find the reasons for this anatomical site distribution.

The microbiological profile of chronic ulcers of the lower limbs has application to general principles of treatment as well as institution-specific guidelines for management [32]. In the present study, *Pseudomonas aeruginosa* was the most frequent gram negative bacteria isolated, whereas gram positive bacteria commonly isolated was *Staphylococcus aureus*. Similar bacterial profile was reported by Lim *et al.*[32]. The study also found that most of the pathogens were multiply resistant to the commonly prescribed antibiotics such as Ampicillin, Augmentin, Cotrimoxazole, Tetracycline, gentamicin, erythromycin, and Ceftriaxone. Similar antimicrobial susceptibility pattern has been reported previously [33]. These findings reflect the widespread and indiscriminate use of antibiotics, coupled with poor patient compliance and self treatment without prescription among African patients [32,33]. The majority of gram negative isolates were sensitive to Meropenem while gram positive being sensitive to Vancomycin; this could be explained by the fact that these antibiotics are relatively rare in the hospital and are more expensive so they are rarely misused.

The prevalence of HIV infection in the present study was 6.7% that is relatively similar to that in the general population in Tanzania (6.5%) [34]. High HIV seroprevalence among patients with CLLUs was reported in a Zimbabwean study [35]. HIV seropositive patients have been reported to have a higher risk of developing postoperative complications and have a greater risk of prolonged hospital stay and mortality [16,18]. HIV infection has been reported to increase the risk of wound sepsis and poor healing [35]. However, in the present study, there were no significant differences in the outcome between patients who are HIV infected and those who are non-HIV infected.

Fungal infections have been reported to be common in chronic lower limb ulcers with the prevalence ranging from 4.5%–50% and [36,37], are also responsible for some chronic lower limb ulcers e.g. Madura foot [38,39]. In the present study, fungal infection was not investigated due to logistic problems. This calls for other authors to investigate on this.

Histopathological examination remains the most important definitive diagnostic procedure, and it should be performed on any suspicious lesion or any chronic non-healing ulcers, especially those with any recent change in appearance or considerable drainage [40]. In the present study, malignant ulcers were histopathologically proven in 8.4% of cases, a figure closely to 10.4% reported by Senet *et al.*[41]. In our study, malignant melanoma was the most frequent histopathological type as previously reported by Chalya *et al.*[42] at the same centre, but at variant with Senet *et al.*[41] who reported squamous cell carcinoma as most common histopathological type. This difference in histopathological type reflects geographic differences in exposure to risk factors for developing malignant ulcers. While solar radiation has been suggested as a major cause of malignant melanoma among Caucasians, many of malignant melanoma among black Africans has been reported to be unrelated to solar exposure since they occur on the unexposed plantar of the foot [43-51]. Higher incidence of malignant melanoma in our study may be attributed to repeated trauma and constant pressure on the weight bearing areas of the foot as shoe-wearing is less frequent among people especially those from rural areas [42,52]. In the present study, Kaposi’s sarcoma ranked third after squamous cell carcinoma. Since the emergence of HIV infection, there has been a steady increase in the prevalence of KS worldwide [53,54]. The rate of HIV infection among patients with Kaposi’s sarcoma in our study was 60%, a figure slightly lower than that reported by Chalya *et al.*[42]. Thus it is obvious that successful HIV control will go a long way to reduce the incidence of this vascular malignancy.

The treatment of chronic lower limb ulcers requires multidisciplinary approach [54,55]. The treatment modalities of chronic lower limb ulcers include surgical treatment (such as wound debridement, wide local excision, split thickness skin graft (STSG) or flap cover, block dissection of the regional nodes and limb amputation in advanced lesions) and non-surgical treatment such daily dressing, compressive bandages and antimicrobial agents bases on drug sensitivity pattern [54,56-58]. In the present study, wound debridement with or without STSG or flap cover was the most common surgical procedure performed which is in keeping with studies done elsewhere [55].

The presence of complications has an impact on the final outcome of patients presenting with chronic lower limb ulcers [21]. Most complications are related to late presentation to hospital following ignorance, treatment at home, cost, poverty, advanced malignancy, premorbid conditions like diabetes mellitus, hypertension, and the treatment choices made and the procedures performed. In the present study a total of 178 complications were recorded in 175 (58.3%) patients, mostly being post operative complications. Of these, surgical site infections (77.5%) was the most common post operative complication followed by recurrent ulceration (11.2%) and skin grafting failure (6.2%). Callam *et al.*[21] reported a similar observation.

The length of hospital stay is an important measure of morbidity in which estimates of length of hospital stay are important for financial matters and accurate early estimates so as to facilitate better financial planning by the payers since it takes long for the chronic lower limb ulcers to heal so increasing the costs as well as seen in other studies as well [16,18]. In this study, the overall mean length of hospital stay was 28.9 days, a figure which is higher than that reported in other studies [59,60]. A mean length of hospital stay of 38.2 days was also reported in Nigerian study [16]. A mean of 36.2 days and 64.2 days were reported in Tanzanian and Nigerian studies respectively [16,24]. Prolonged LOS in our study was observed in patients with diabetic foot ulcers and in patients who required surgical treatment.

In this study, the mortality rate was 4.3% which is relatively lower than that reported in other studies [16]. Mortality rate in the present study was attributed to complications of diabetes mellitus, hypertension, HIV infection and advanced malignancy. The causes of death in our study is at variant with a Nigerian study which reported anemic heart failure, septicemia and multiple organ failure as causes of death [16]. Addressing these factors responsible for mortality in our patients is mandatory to be able to reduce mortality associated with chronic lower limb ulcers.

In this study, complete healing at discharge from the hospital was achieved in more than 90% of the patients, which is comparable with other studies [16,61]. This is satisfactorily acceptable to both the patient and the surgical team.

Self discharge by patient against medical advice is a recognized problem in our setting and this is rampant, especially amongst patients with chronic lower limb ulcers [62]. In the present study discharge against medical advice was noted in 0.7% of cases. Discharge against medical advice in our study is attributed to patients feeling well enough to leave and dissatisfaction with treatment received.

Poor follow up visits after discharge from hospitals remain a cause for concern in most developing countries [63]. These issues are often the results of poverty, long distance from the hospitals and ignorance. In the present study, only 33.1% of patients were available for follow up at three months, the reasons for low follow up rate at our study may be attributed to long distance from the hospital, lack of funds for transport and feeling of being cured.

Delay in getting histopathological results was the major limitation in this study and this might have affected the treatment outcome of patients who needed this confirmatory diagnostic investigation for definitive treatment.

## Conclusion

Chronic lower limb ulceration remains a major public health problem in this part of Tanzania. Traumatic ulcers are the most common type of chronic lower limb ulcers. The majority of patients in our environment present late when the disease is already in advanced stages predisposing them to increased risk of recurrence, malignant change and limb amputation. Early recognition and aggressive treatment of the acute phase of chronic lower limb ulcers at the peripheral hospitals and close follow-up are urgently needed to improve outcomes of these patients in this environment. Further study looking at the factors associated with late presentation to tertiary health facilities is highly recommended. A population based study is highly needed to be able to assess the better picture of the magnitude of the problem in this region.

## Competing interests

The authors declare that they have no competing interests.

## Authors’ contributions

FM conceived the study and did the literature search, participated in data analysis, writing of the manuscript and editing. MDM and BBK participated in the literature search, writing of the manuscript and editing. SEM participated in writing of the manuscript, editing and performed the microbiological analysis. PFR participated in writing of the manuscript, editing and performed the pathological work up. PLC participated in writing of the manuscript, editing, data analysis and submission of the manuscript. JMG coordinated the write-up, editing and supervised the study. All the authors read and approved the final manuscript.

## Pre-publication history

The pre-publication history for this paper can be accessed here:

http://www.biomedcentral.com/1471-5945/12/17/prepub
